# αB-crystallin expression in breast cancer is associated with brain metastasis

**DOI:** 10.1038/npjbcancer.2015.14

**Published:** 2015-10-21

**Authors:** K David Voduc, Torsten O Nielsen, Charles M Perou, J Chuck Harrell, Cheng Fan, Hagen Kennecke, Andy J Minn, Vincent L Cryns, Maggie C U Cheang

**Affiliations:** 1 Department of Radiation Oncology, University of British Columbia, Vancouver, BC, Canada; 2 Department of Pathology and Laboratory Medicine, University of British Columbia, Vancouver, BC, Canada; 3 Department of Genetics, Lineberger Comprehensive Cancer Center, University of North Carolina at Chapel Hill, Chapel Hill, NC, USA; 4 Department of Pathology, Virginia Commonwealth University, Richmond, VA, USA; 5 Division of Medical Oncology, British Columbia Cancer Agency, University of British Columbia, Vancouver, BC, Canada; 6 Department of Radiation Oncology, Abramson Family Cancer Research Institute, Perelman School of Medicine, University of Pennsylvania, Philadelphia, PA, USA; 7 Department of Medicine, University of Wisconsin Carbone Cancer Center, University of Wisconsin School of Medicine and Public Health, Madison, WI, USA; 8 Division of Clinical Studies, Clinical Trials and Statistics Unit (ICR-CTSU), Institute of Cancer Research, London, UK

## Abstract

**Background/objectives:**

The molecular chaperone αB-crystallin is expressed in estrogen receptor, progesterone receptor and human epidermal growth factor receptor-2 ‘triple-negative’ breast carcinomas and promotes brain and lung metastasis. We examined αB-crystallin expression in primary breast carcinomas with metastatic data to evaluate its association with prognosis and site-specific metastases.

**Methods:**

αB-crystallin gene (*CRYAB*) expression was examined using publically available global-gene expression data (*n*=855 breast tumors) with first site of distant metastasis information (‘855Met’). αB-crystallin protein expression was determined by immunohistochemistry using the clinically annotated British Columbia Cancer Agency (BCCA) tissue microarray (*n*=3,987 breast tumors). Kaplan–Meier and multivariable Cox regression analyses were used to evaluate the prognostic value of αB-crystallin. Multivariable logistic regression analysis was used to evaluate risks of αB-crystallin and other markers for site of metastasis.

**Results:**

In the 855Met data set, αB-crystallin gene (*CRYAB)* expression was an independent predictor of brain as the first distant site of relapse (hazards ratio, HR=1.2, (95% confidence interval, CI 1.0–1.4), *P*=0.021). In the BCCA series, αB-crystallin protein expression was an independent prognostic marker of poor breast cancer-specific survival (HR=1.3, (95% CI 1.1–1.6), *P*=0.014). Among patients with metastases, αB-crystallin was the strongest independent predictor of brain metastasis (odds ratio, OR=2.99 (95% CI 1.83–4.89), *P*<0.0001) and the only independent predictor of brain as the first site of distant metastasis (OR=3.15 (95% CI 1.43–6.95), *P*=0.005). αB-crystallin was also associated with worse survival (3.0 versus 4.7 months, *P*=0.007).

**Conclusions:**

αB-crystallin is a promising biomarker to identify breast cancer patients at high risk for early relapse in the brain, independent of estrogen receptor and human epidermal growth factor receptor-2 status.

## INTRODUCTION

Over the past 15 years, gene expression studies in breast cancer have led to more accurate prognostic tools and to a greater understanding of the molecular heterogeneity of breast cancer.^1–5^ Basal-like breast carcinomas have emerged as a distinctive molecular subtype defined by expression of genes characteristic of basal epithelial cells that overlap in large part with estrogen receptor (ER), progesterone receptor (PR), and human epidermal growth factor receptor-2 (HER2) ‘triple-negative’ breast cancers (TNBC).^6,7^ More recent studies point to molecular heterogeneity within the triple-negative subgroup with potential prognostic and therapeutic implications.^8–10^ Overall, basal-like tumors are associated with a higher risk of recurrence following conventional adjuvant treatment and poor survival.^4,11,12^ Furthermore, basal-like tumors have a characteristic pattern of distant metastasis to the lungs and brain with infrequent relapse in bone.^13–15^ Although lung and brain metastasis gene signatures have been identified,^16,17^ the functional contribution of individual genes to site-specific metastasis in this subtype is poorly understood. Clearly, the identification of pathogenic drivers of brain and lung metastasis in this subtype could lead to biomarkers to help identify women at high risk for relapse at these sites.

αB-crystallin is a widely expressed member of the small heat shock protein family that protects cells from stress by its dual function as a molecular chaperone to preserve proteostasis and as a cell death antagonist that inhibits caspase-3 activation and oxidative stress.^18–22^ αB-crystallin is commonly expressed in basal-like and TNBCs and its expression correlates with resistance to neoadjuvant chemotherapy and poor survival.^23–26^ More recently, αB-crystallin has been demonstrated to inhibit extracellular matrix detachment-induced apoptosis (‘anoikis’), enhance penetration through an endothelial/astrocyte co-culture model of the blood–brain barrier *in vitro*, and promote lung and brain metastasis in orthotopic models of TNBC in immunodeficient mice.^27,28^ In addition, αB-crystallin is frequently expressed in brain metastases and its expression in primary breast carcinomas is associated with poor overall survival and poor survival after brain metastasis in a cohort of 76 women with breast cancer brain metastasis.^28^ Collectively, these findings point to a key pathogenic role of αB-crystallin in lung and brain metastasis in TNBC.

Given the recent evidence supporting a prometastatic function for αB-crystallin in TNBC, we postulated that αB-crystallin might have utility as a prognostic biomarker to predict the risk of disease progression in TNBC patients. To this end, we examined the prognostic value of αB-crystallin (gene and protein) expression in breast cancer using (1) publically available gene expression data from multiple breast cancer cohorts, which included sites of first distant metastasis, and (2) a large breast cancer tissue microarray (TMA) linked to long-term outcomes, including metastatic sites. Using these data sets, we confirmed the association between αB-crystallin protein expression and basal-like phenotype and poor survival in univariable and multivariable models. Moreover, we observed that expression of the αB-crystallin gene (*CRYAB*) was associated with brain as a first site of distant relapse, while αB-crystallin protein expression was associated with the development of brain metastasis (first site or any occurrence) and poor survival after diagnosis of brain metastasis. Collectively, our findings suggest that αB-crystallin (gene and/or protein) expression may be of clinical utility as a biomarker to identify women at high risk for brain metastasis.

## MATERIALS AND METHODS

### Human breast tumor gene expression analysis

Publicly available global-gene expression profiles of 855 tumors were downloaded from four studies (GSE2034, GSE12276, GSE2603, and the NKI295), and combined using Distance Weighted Discrimination to remove the systematic biases present in different microarray data sets.^29^ The four tumor data sets contained the sites of first distant relapses and time to relapse, and will be referred as ‘855Met’ in this current study. The combined clinical data can be found at the University of North Carolina microarray database.^30^ Gene expression data were row (gene) median centered and column (sample) standardized (s.d.=1), respectively. Tumors were assigned into one of the intrinsic subtypes using the PAM50 classifier.^2^

### British Columbia cancer agency cohort

The British Columbia cancer agency (BCCA) cohort includes 3,987 formalin-fixed paraffin-embedded specimens from female patients with newly diagnosed, invasive breast cancer referred to BCCA from 1986 to 1992 that were used to construct a large TMA linked to detailed clinicopathological data and long-term outcomes (median follow-up of 12 years).^12,15^ Most patients were treated with adjuvant systemic therapy according to provincial cancer management guidelines. The BC Cancer Agency Research Ethics Board approved all biomarker studies on tissue specimens, and associated clinical and outcomes data correlation.

### Immunohistochemistry and TMAs

TMAs were constructed from tissue blocks as described.^8^ αB-crystallin protein expression was evaluated by immunohistochemistry (IHC) using a mouse monoclonal antibody (1:200 dilution; ADI-SPA-222; Stressgen Biotechnologies/Enzo Life Sciences, Farmingdale, NY, USA) as described previously and was scored using an established, previously published cutoff, whereby tumors were considered positive for αB-crystallin if there was any staining above background levels.^23^ αB-crystallin protein expression was scored by a pathologist (TON) who was blinded to clinical outcomes. Samples with <50 tumor cells present in the TMA core were excluded from the analysis. TMAs were digitally scanned, and the images are available for public access (http://www.gpecimage.ubc.ca/tma/web/viewer.php; username: CRYAB4000; password: CRYAB4000). Breast cancer subtypes were defined using a previously validated immunopanel of six biomarkers including ER, PR, HER2, Ki67, epidermal growth factor receptor (EGFR), and cytokeratin 5/6 (CK5/6).^8,31^ In brief, tumors were classified as Luminal A if ER and/or PR positive, and HER2 negative, and Ki67 low (<14%); Luminal B if ER and/or PR positive, and HER2 negative, and Ki67 high (⩾14%); Luminal-HER2 if ER and/or PR positive, and HER2 positive; HER2 enriched if ER and PR negative and HER2 positive; Basal-like if negative for all ER, PR, and HER2 but expressing EGFR and/or CK5/6; and as Triple-negative non-basal if negative for ER, PR, HER2, EGFR, and CK5/6.

### Statistical analysis

Univariable survival estimates were calculated using the Kaplan–Meier method, and survival differences were estimated using log-rank tests. Due to the retrospective nature of the gene expression and IHC studies, the sample size for each study was not pre-specified. The primary endpoint for the BCCA cohort is breast cancer-specific survival (BCSS). Multivariable Cox regression analyses were used to test the independent prognostic value of αB-crystallin. Smoothed plots of weighted Schoenfeld residuals were used to test proportional hazard assumptions. The association of αB-crystallin expression with intrinsic subtypes was assessed using the *χ*^2^-test. Multivariable logistic regression analysis was used to evaluate the association of αB-crystallin and other clinicopathological markers for site of metastasis.

## RESULTS

### αB-crystallin gene (*CRYAB*) expression is associated with distant relapse in basal-like tumors

To investigate whether expression of the αB-crystallin gene (*CRYAB*) is associated with survival and site-specific metastasis, we interrogated publicly available gene expression data of 855 breast tumors from four studies with available information regarding the first site of distant metastasis (‘855Met’). Within the 855Met data set, there were 188 basal-like tumors defined by gene expression using the PAM50 classifier. Within this subset of basal-like tumors, *CRYAB* expression was significantly associated with an increased risk of distant relapse by multivariable Cox regression analysis adjusted for ER status, age at diagnosis and nodal status (Table 1, hazards ratio=1.1 (95% confidence interval, CI 1.0–1.2), *P*=0.045). This suggested that *CRYAB* gene expression could be a potential biomarker for metastatic behavior of basal-like tumors.

### *CRYAB* expression is associated with brain as the first site of distant relapse

Given the recent study linking αB-crystallin to breast cancer brain metastasis,^28^ we examined the relationship between *CRYAB* expression and brain metastasis as the first distant relapse. In the 855Met data set, 376 patients developed distant relapse: 49 of these patients had brain metastasis as the first site of relapse. In the multivariable Cox regression analysis for brain as a first site of distant relapse of using all available cases in this cohort (852 tumors), *CRYAB* expression was significantly associated with earlier development of brain relapse, independent of standard clinicopathological variables, including the PAM50-defined intrinsic subtypes (Table 2, hazards ratio=1.2 (95% CI 1.0–1.4), *P*=0.021). Interestingly, although intrinsic subtypes were still significantly associated with distant relapse to brain, the basal-like subtype was not independently significant, when compared with Luminal A tumors, in the multivariable model including *CRYAB*. Analysis of the subgroup of 376 patients who developed distant recurrences revealed that *CRYAB* expression was significantly associated with increased likelihood of developing brain metastasis (odds ratio: 1.2 (95% CI 1.0–1.3), *P*=0.016). However, this latter association was not independent of gene expression-based intrinsic subtypes in the multivariable logistic regression analysis (*P*=0.08) of this smaller subset of patients. Collectively, these gene expression analyses indicate that *CRYAB* expression is associated with increased risk of disease progression in TNBC and with brain metastasis as a first site of distant relapse in the entire cohort.

### αB-crystallin protein expression is associated with the basal-like phenotype and poor survival

To validate and extend our observations from gene expression studies, we interrogated the BCCA TMA of 3,987 breast tumors for the expression of αB-crystallin protein by IHC. The clinicopathological characteristics and treatment regimens for this cohort are indicated (Table 3). Scoring of αB-crystallin expression was possible for 3,235 cases. Of these, 359 (11%) were positive for αB-crystallin expression. All the stained TMAs were digitally scanned, and primary image data are available for public access: http://www.gpecimage.ubc.ca/tma/web/viewer.php; (username: CRYAB4000; password: CRYAB4000). Using the six biomarker immunopanel to subclassify each breast tumor,^8,31^ a molecular subtype was assigned to 3,009 of the 3,235 breast tumors with αB-crystallin IHC data. αB-crystallin was infrequently expressed in Luminal A tumors (54 of 1312, 4.1%), Luminal B tumors (33 of 766, 4.4%), Luminal–HER2^+^ tumors (8 of 199, 4.0%) or HER2^+^ tumors (18 of 221, 8.1%). In contrast, αB-crystallin was highly expressed in basal-like tumors defined by triple-negative status and expression of basal markers CK5/6 or EGFR (170 of 309, 55%) and to a lesser extent in triple-negative tumors that lacked expression of these basal markers (47 of 202, 23%). The differential distribution of αB-crystallin expression among the various molecular subtypes of breast cancer was highly statistically significant (*P*<0.0001 by *Χ*^2^-test). These findings are consistent with prior reports specifically linking αB-crystallin expression to basal-like breast carcinomas/TNBCs.^23–26,28,32,33^

**Figure 1:**
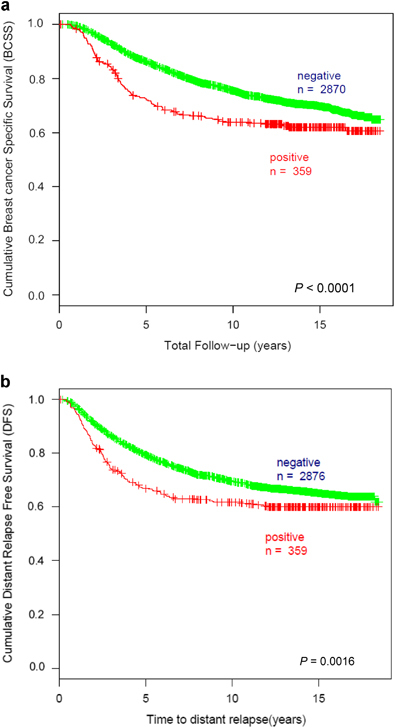
αB-crystallin expression is associated with worse breast cancer-specific survival (BCSS) and distant relapse-free survival (DFS) in univariable analyses. (**a**) Kaplan–Meier analysis of BCSS based on αB-crystallin expression. 10-year BCSS was 64% for αB-crystallin-positive cases (*n*=359, number of events=133) versus 75% for αB-crystallin-negative cases (*n*=2,870, number of events=809), *P*<0.0001 by log-rank test. (**b**) Kaplan–Meier analysis of DFS based on αB-crystallin expression. 10-year DFS was 62% for αB-crystallin-positive cases (*n*=359, number of events=139) versus 70% for αB-crystallin-negative cases (*n*=2876, number of events=915), *P*=0.0016 by log-rank test.

In univariable analysis among all breast cancer subtypes, αB-crystallin was a statistically significant marker of poor prognosis. BCSS at 10 years was 64% in αB-crystallin-positive cases versus 75% in αB-crystallin-negative cases (Figure 1a, *P*<0.0001 by log-rank test). Distant relapse-free survival at 10 years was 62% in αB-crystallin-positive cases versus 70% in αB-crystallin-negative cases (Figure 1b, *P*=0.0016 by log-rank test). αB-crystallin had independent prognostic significance for BCSS (Table 4, hazards ratio=1.3 (95% CI 1.1–1.6), *P*=0.014) in a multivariable Cox model adjusting for patient age, tumor grade, systemic therapy, ER, and HER2 status. In this model, patient age, tumor grade, lymph node status, tumor size, and HER2 status were also independently prognostic for BCSS. These findings validate our prior results from a much smaller cohort of breast cancer patients (*n*=438) that αB-crystallin protein expression is an independent prognostic biomarker of BCSS.^23^

### αB-crystallin expression is associated with site-specific brain metastasis

1,054 (33%) of patients in the BCCA cohort with available αB-crystallin IHC staining developed metastatic breast cancer to one or more organs, including 148 (4.5%) patients who developed brain metastases. Among the patients with metastatic disease, αB-crystallin expression was significantly associated with brain metastasis: 12% of patients with αB-crystallin-negative metastatic breast cancer had brain metastasis, in contrast to 33% of patients with αB-crystallin-positive cancer (*P*<0.0001 by Fischer's exact test). In a multivariable logistic regression model including only patients with metastatic breast cancer, αB-crystallin was the strongest independent predictor of brain metastasis (odds ratio=2.99 (95% CI 1.83–4.89), *P*<0.0001); ER and HER2 status were also independent predictors of brain metastasis (Table 5). Multivariable logistic regression, relating tumor size, nodal status, tumor grade, systemic therapy, ER, HER, and αB-crystallin to the development of brain metastasis as the first site of distant relapse found that αB-crystallin status was the only independent predictor for brain metastasis as the first site of distant relapse (odds ratio=3.15 (95% CI 1.43–6.95), *P*=0.005). αB-crystallin was also associated with a shorter time interval between initial diagnosis of breast cancer and the subsequent diagnosis of brain metastasis (median time to brain metastasis=2.7 years versus 4.2 years, *P*<0.0001 by log-rank test). Following the diagnosis of brain metastasis, patients with αB-crystallin-positive metastatic breast cancer had worse survival (median survival=3.0 months versus 4.7 months, *P*=0.007 by log-rank test). In addition, αB-crystallin expression was associated with lower risk of liver metastasis (odds ratio=0.53, *P*=0.009). However, there was no significant association between αB-crystallin expression and lung or bone metastases. Collectively, these results indicate that αB-crystallin protein expression is an independent predictor for the development of brain metastasis (first site or any occurrence) and is associated with poor survival after diagnosis of brain metastasis relative to metastatic αB-crystallin-negative breast cancer.

## DISCUSSION

HER2-positive and basal-like tumors/TNBC have a distinctive pattern of organ-specific distant metastasis that includes brain metastasis, a debilitating event that typically follows a rapidly fatal course.^13–15,34^ Indeed, the prevalence of brain metastases among patients with metastatic TNBC has been reported as high as 46% with median survival after diagnosis of 4.9–6.6 months.^35,36^ Moreover, the brain is the first site of distant metastasis in 4.7% of patients with early-stage TNBC.^37^ Although HER2 has been previously linked to the pathogenesis of brain metastasis, as discussed further below, αB-crystallin has only recently been reported to have a pathogenic role in brain metastasis in TNBC models.^28,38–40^ Consistent with this pathogenic role, we have demonstrated that αB-crystallin gene (*CRYAB*) expression is associated with distant relapse in TNBC patients and with earlier development of brain relapse as a first distant site, independent of standard clinicopathological variables and PAM50 subtyping in the entire cohort. Moreover, αB-crystallin protein expression in the primary breast tumor was an independent prognostic biomarker of poor BCSS and was associated with a 33% risk of brain metastasis among patients in the BCCA cohort who developed metastatic disease. This is comparable to the high incidence of brain metastasis observed in HER2-positive metastatic breast cancer (25–40%).^38^ Notably, both HER2 and αB-crystallin were independent predictors of brain metastasis (any occurrence) in multivariate analyses. However, αB-crystallin expression was the strongest predictor of brain metastasis (any occurrence) and the only independent predictor of brain metastasis as the first site of distant relapse. These findings suggest that αB-crystallin (gene and/or protein) expression in primary breast cancers may be a useful biomarker to identify patients at greater risk for developing brain metastasis. Because we observed that αB-crystallin is preferentially expressed in basal-like tumors (55%) and rarely in HER2-positive tumors (8.1%), consistent with prior reports, our findings suggest that the αB-crystallin expression status may be particularly informative among TNBC patients to help identify individuals at greater risk for early or first-site brain relapse.^23–26,28,32,33,37^

We have also demonstrated that αB-crystallin protein expression is associated with a shorter time interval between the breast cancer diagnosis and the development of brain metastasis (2.7 years versus 4.2 years, *P*<0.0001 by log-rank test) and shorter survival after diagnosis of brain metastasis (3.0 months versus 4.7 months, *P*=0.007 by log-rank test). These results are particularly striking given the poor survival of patients who developed brain metastasis. Even among this subset of poor-prognosis patients, αB-crystallin expression was associated with significantly shorter survival after diagnosis of brain metastasis. Our findings are consistent with a recent report from a cohort of 76 patients with breast cancer who developed brain metastasis.^28^ In that cohort, αB-crystallin expression was associated with inferior survival after breast cancer diagnosis (1.4 vs. 4.7 years, *P*=0.0002) and survival after brain metastasis diagnosis (0.13 vs. 0.91 years, *P*=0.001). Collectively, these results point to αB-crystallin expression in primary breast cancers as a promising biomarker to identify patients at high risk for early relapse in the brain and poor survival after diagnosis. As such, these patients could be enrolled in clinical trials to evaluate preventive agents or modalities for brain metastasis, particularly as less toxic options become available. Indeed, with further validation, αB-crystallin expression testing could be incorporated as one of the pre-planned biomarker tests for contemporary biomarker-driven trials in patients with metastatic TNBC with extracranial disease to determine optimal surveillance and treatment approaches. The importance of validating αB-crystallin as a biomarker for brain metastasis is underscored by a recent study in which a promising three-gene signature for the development of early brain metastasis in HER2-positive breast cancer failed subsequent validation.^41^

Intriguingly, the observed association of αB-crystallin expression in primary breast carcinomas and brain metastasis is biologically plausible in the light of recent data from cellular assays and murine models pointing to a pathogenic role for αB-crystallin in breast cancer brain metastasis. αB-crystallin promotes adhesion to human brain microvascular endothelial cells, transendothelial migration and penetration through an human brain microvascular endothelial cell or primary human astrocyte co-culture blood–brain barrier model.^28^ Furthermore, overexpression of αB-crystallin in human TNBC cells enhances brain metastasis in an orthotopic model of metastatic TNBC characterized by widespread distant metastases, whereas silencing αB-crystallin inhibits brain metastasis in a second orthotopic TNBC model.^28^ It is tempting to speculate that the enhanced adhesion to human brain microvascular endothelial cells and blood–brain barrier penetration *in vitro*, two critical steps in brain metastasis,^40^ could provide a biological rationale for the observed robust association between αB-crystallin expression in primary breast cancers and increased risk of developing brain metastasis, particularly aggressive brain metastases that develop rapidly after breast cancer diagnosis. Consistent with this idea, αB-crystallin is commonly expressed in brain metastases from patients with αB-crystallin-positive breast carcinomas, while some αB-crystallin-positive brain metastases develop from primary breast cancers that lack αB-crystallin expression.^28^ αB-crystallin also promotes resistance to matrix detachment-induced apoptosis (anoikis), a hallmark of metastatic carcinoma cells, which would be expected to promote circulating tumor cell survival.^27,42^ Although αB-crystallin promotes lung metastasis in some orthotopic TNBC models,^27^ our IHC data point to a specific association between αB-crystallin expression in breast tumors and brain metastasis; there was no correlation with lung metastasis in the BCCA cohort. These latter findings suggest that preclinical murine models may not accurately recapitulate all aspects of the metastatic cascade in patients. Nevertheless, our clinical data point to a robust association between αB-crystallin and early metastasis to the brain that is highly congruent with its recently delineated pathogenic role in brain metastasis.

In summary, we present data from multiple breast cancer cohorts with metastatic site data demonstrating that αB-crystallin gene (*CRYAB*) expression in primary breast carcinomas is an independent predictor for the development of brain as the first site of distant relapse. We have also demonstrated that αB-crystallin protein expression in primary breast tumors is an independent prognostic biomarker for poor BCSS, early site-specific metastasis to the brain, and worse survival after the diagnosis of brain metastasis. We plan to examine the correlation between αB-crystallin gene and protein expression in appropriate breast cancer cohorts to determine whether one is a better biomarker than the other or whether they are equivalent. Collectively, our findings point to αB-crystallin (gene and/or protein expression) as a promising biomarker to identify patients at high risk for early relapse in the brain who could be enrolled in clinical trials to evaluate preventive strategies.

## Figures and Tables

**Table 1:** Multivariable Cox regression analysis for the development of any distant relapse in 188 patients with basal-like breast tumors in the 855Met cohort

*Basal-like tumors*^a^	*Hazard ratio to distant relapse (95% CI)*	P*-value*
*CRYAB*	1.1 (1.0–1.2)	0.045
ER positive (*n*=29) vs. negative (*n*=159)	1.0 (0.6–1.8)	0.96
Age (continuous variable)	1.0 (0.97–1.0)	0.43
Nodal status positive (*n*=28) vs. negative (*n*=160)	0.5 (0.3–1.0)	0.066

Abbreviations: CI, confidence interval; ER, estrogen receptor.

a *n*=188, events=89.

**Table 2:** Multivariable Cox regression analysis for the development of brain as the first site of distant relapse among 852 patients with complete clinicopathological data in the 855Met cohort

*Breast tumors*^a^	*Hazard ratio to brain mets as first site (95% CI)*	P*-value*
*CRYAB*	1.2 (1.0–1.4)	0.021
ER positive (*n*=596) vs. negative (*n*=256)	0.4 (0.2–0.9)	0.025
Nodal status positive (*n*=158) vs. negative (*n*=694)	1.0 (0.5–2.0)	0.9
Age (continuous)	0.98 (0.9–1.0)	0.078
PAM50		0.036
LumA	1	
LumB	4.7 (1.7–13)	0.0031
HER2 enriched	2.9 (0.9–9.3)	0.066
Basal like	1.3 (0.4–4.7)	0.66
Normal like	1.7 (0.5–5.6)	0.39

Abbreviations: CI, confidence interval; ER, estrogen receptor; HER2, human epidermal growth factor receptor-2; LumA, luminal A; LumB, luminal B.

a *n*=852, brain metastasis events=49.

**Table 3:** Clinicopathological characteristics and treatment regimens of the 3,987 patients in the BCCA cohort

	N	*%*
*Age (years)*		
<40	294	7
40–49	843	21
50–65	1,423	36
>65	1,427	36
		
*Grade*		
1	209	6
2	1,562	41
3	2,037	54
NA	179	
		
*Menstrual status at referral*		
Pre-menopausal	1,178	30
Post-menopausal	2,717	70
NA	92	
		
*Nodal status*		
Negative	2,265	57
Positive	1,714	43
NA	8	
		
*Lymphovascular invasion*		
Negative	2,105	55
Positive	1,709	45
NA	173	
		
*Tumor size (cm)*		
≤2	2,077	52
2–5	1,665	42
>5	221	6
NA	24	
		
*Initial Adjuvant Systemic Therapy*		
None	1,676	42
Tamoxifen only	1,274	32
Chemotherapy only (AC, FAC or CMF)	725	18
Tamoxifen and chemotherapy	296	8
Other	16	

Abbreviations: AC, adriamycin and cyclophosphamide; BCCA, British Columbia Cancer Agency; CMF, cyclophosphamide, methotrexate, and 5-fluorouracil; FAC, 5-fluorouracil and AC; NA, not available.

**Table 4:** Cox multivariable analysis for breast cancer-specific survival (BCSS) in the BCCA cohort^a^

*Variables*	*HR*	*95% CI*	P*-value*
Age at diagnosis, years			0.022
>55 (*n*=1,790)	1		
<40 (*n*=239)	1.2	0.91–1.5	0.22
40–55 (*n*=946)	0.87	0.72–1.0	0.12
			
Grade	1.5	1.3–1.8	
3 (*n*=1,643) vs. 2/1 (*n*=1,332)			<0.0001
			
Nodal status	2.6	2.1–3.0	
Positive (*n*=1,686) vs. negative (*n*=1,289)			<0.0001
			
Tumor size, cm			<0.0001
⩽2 (*n*=1,545)	1		
2–5 (*n*=1,290)	1.6	1.4–1.8	<0.0001
>5 (*n*=140)	2.2	1.6–2.8	<0.0001
			
*Initial systemic therapy*			
Any hormonal (*n*=1,128) vs. none (*n*=1,847)	0.88	0.74–1.1	0.16
Any chemo (*n*=773) vs. none (*n*=2,202)	0.86	0.69–1.1	0.14
			
*Biomarkers*			
ER-positive (*n*=2,148) vs. negative (*n*=827)	0.89	0.75–1.1	0.18
HER2-positive (*n*=414) vs. negative (*n*=2,561)	1.4	1.2–1.7	<0.0001
αB-crystallin-positive (*n*=332) vs. negative (*n*=2,643)	1.3	1.1–1.6	0.014

Abbreviations: BCCA, British Columbia Cancer Agency; CI, confidence interval; ER, estrogen receptor; HER2, human estrogen receptor-2; HR, hazards ratio.

a *n*=2,972 cases with complete covariate data, also excluding 3 cases censored before the first event.

**Table 5:** Multivariable logistic regression analysis for the development of any brain metastasis and brain as the first site of relapse in the BCCA cohort

	*Odds to any brain mets (95% CI), (events=141)*	P*-value*	*Odds to brain as first distant site (95% CI), (events=46)*	P*-value*
*Tumor size*				
⩽2 cm (*n*=375)	1		1	
2–5 cm (*n*=525)	1.13 (0.76–1.69)	0.54	0.70 (0.37–1.33)	0.28
>5 cm (*n*=69)	0.39 (0·13–1·18)	0.095		
				
*Nodal status*				
Negative (*n*=374)	1		1	
Positive (*n*=595)	0.53 (0·34–0·82)	0.005	0.55 (0.28–1.08)	0.082
				
*Grade*				
1 or 2 (*n*=336)	1		1	
3 (*n*=633)	1.2 (0·76–1·86)	0.441	1.19 (0.57–2.48)	0.65
				
*Any initial chemo*				
No (*n*=648)	1		1	
Yes (*n*=321)	1.46 (0.92–2.30)	0.11	0.36 (0.19–0.71)	0.003
				
*Any initial hormonal*				
No (*n*=556)	1		1	
Yes (*n*=413)	0.79 (0.50–1.24)	0.3	0.91 (0.45–1.84)	0.79
				
*ER*				
Negative (*n*=316)	1		1	
Positive (*n*=653)	0.58 (0.37–0.91)	0.018	0.58 (0.28–1.24)	0.16
				
*HER2*				
Negative (*n*=783)	1		1	
Positive (*n*=186)	1.72 (1.08–2.73)	0.023	1.88 (0.89–3.96)	0.096
				
*αB-crystallin*				
Negative (*n*=842)	1		1	
Positive (*n*=127)	2.99 (1.83–4.89)	<0.0001	3.15 (1.43–6.95)	0.005

Abbreviations: BCCA, British Columbia Cancer Agency; CI, confidence interval; ER, estrogen receptor; HER2, human epidermal growth factor receptor-2.
